# Establishing ‘normal’ career longevity in professional footballers allows comparison to that of players with injuries and surgery

**DOI:** 10.1002/ksa.12722

**Published:** 2025-06-12

**Authors:** Mary Jones, Arman Motesharei, Simon V. Ball, J. Samuel Church, James D. F. Calder, Andy Williams

**Affiliations:** ^1^ Fortius Clinic London UK; ^2^ FIFA Medical Centre of Excellence London UK; ^3^ Imperial College London

**Keywords:** ACL, career longevity, elite sports, football (soccer)

## Abstract

**Purpose:**

To determine the ‘normal’ career longevity of male professional footballers and the factors that affect this in order to provide a baseline against which career longevity after injury can be measured. To demonstrate how these results can be used by comparing them with published career longevity rates after anterior cruciate ligament reconstruction (ACLR).

**Methods:**

Match play data for the entire careers of 4117 male footballers playing in the top four English football leagues between 1992 and 2023 was utilised. Players were grouped into ability levels and their “still playing” rates, and career trajectory tracked according to age. Univariate and multivariate analyses were used to determine differences by playing position and ability and Kaplan–Meier survival curves were generated. The findings were compared with published results after ACLR according to the comparable age and league level.

**Results:**

Goalkeepers had a longer overall career length than outfield players (12.4 ± 4.9 vs. 11.6 ± 4.7 years, *p* = <0.01).Outfield players in the English Premier League (EPL) and those also playing internationally (EPL + I) play for longer overall and longer at their highest level (14.8 ± 3.3 and 7.5 ± 5.0 years) than lower league players (League 2: 6.2 ± 4.1 and 2.9 + 2.2 years). At 5 years, EPL + I and EPL outfield players have a 60% and 40% probability of continuing to play at their highest level respectively compared to less than 20% in The Championship and below. At 10 years this is 40% and 18% respectively compared to <2% in the lower leagues. 'Still playing' rates after ACLR are up to 12.9% lower than average for 30 year old footballers.

**Conclusion:**

Career duration in footballers is affected by the position played and ability level. Career longevity and performance data is provided in a usable format for easy comparisons with studies reporting career longevity outcomes in professional footballers.

**Level of Evidence:**

Level III, retrospective cohort study.

**Abbreviations**
ACL: anterior cruciate ligament
ACLR: anterior cruciate ligament reconstruction
C: championship
EFL: English Football League
EPL: English Premier League
EPL + I: English Premier League and International
FBREF: actual name of site, not an abbreviation
L1: League 1
L2: League 2
RTP: return to play
SD: standard deviation
UEFA: Union of European Football Associations

## INTRODUCTION

Football is the most popular sport in the world, and in 2021 there were nearly 130,000 professional players and over 4400 professional clubs worldwide [37]. It is estimated that 8.1 injuries are sustained in professional football for every 1000 h of exposure, with the match play injury rate being nearly 10 times higher when compared to practice and training [12, 23]. Following any injury, the main concerns of the player and their entourage are: will they be able to return to play (RTP), will it affect their performance, and what long term effect will it have on their career? However, although failure to RTP, a reduction in performance, and reduced longevity of career occurs after musculoskeletal injuries [2, 4, 15, 17, 22, 28, 34, 35], it is not known how these compare to the natural reduction in playing rates and levels of professional footballers over time from simple attrition.

Anterior cruciate ligament (ACL) injuries are common in footballers [13, 16, 32]. Several studies have investigated career longevity after ACL reconstruction (ACLR) [2, 6, 14, 25, 27, 28, 34, 35], with between 30% and 84% of athletes 'still playing' at 5 years after surgery. Comparisons of elite footballers following ACLR with matched, uninjured controls have demonstrated reduced career duration and performance levels after ACLR [2, 6, 27]. However, the uninjured control groups used by Borque et al. [6] and Niederer et al. [27] also demonstrated reduced playing rates over time, with only 92% and 80% of footballers respectively, 'still playing' at 5 years.

There is now much publicly available data on sport‐specific internet sites which document performance metrics such as match appearances and minutes played. Although there are recent concerns that injury data in these public sites has been shown to be inaccurate [8, 11, 19], it can nevertheless provide a useful source for determining injured players' pre‐ and post‐injury performance levels when it is used in conjunction with information from the patient's medical records [29].

Ideally, when monitoring the outcomes of orthopaedic interventions, players' post‐treatment performance would be compared with the data from matched uninjured comparisons, but this is not always feasible. However, a better understanding of the average longevity and performance levels for footballers, including how they vary with age and ability would allow meaningful comparisons with injured players to be easily made, whatever the injury or treatment, and so determine the impact of injuries, and their treatments, on career longevity and performance level. Therefore, the purpose of the present study is to determine ‘average’ career longevity for male footballers playing professional football in England. The secondary aims are to demonstrate how this varies according to age, playing position, and level played. To illustrate the usefulness of this data, another aim is to evaluate changes in career longevity after anterior cruciate ligament reconstruction.

## METHODS

All players who had three or more match appearances in the top four English football leagues (English Premier League [EPL], Championship [C], League One [L1] and League Two [L2]) between the 2005/06 season and the 2009/10 season were eligible for inclusion in the study. Football match appearance data for these players' entire senior careers, which spanned all seasons between 1992/93 and 2022/23, was utilised in the study. Of the 4257 players eligible for inclusion 140 were excluded: three as their performance data was not fully available and 137 who played less than three senior matches leaving 4117 footballers included in the present study. Football match appearance data was retrieved from www.fbref.com.

**Figure 1:**
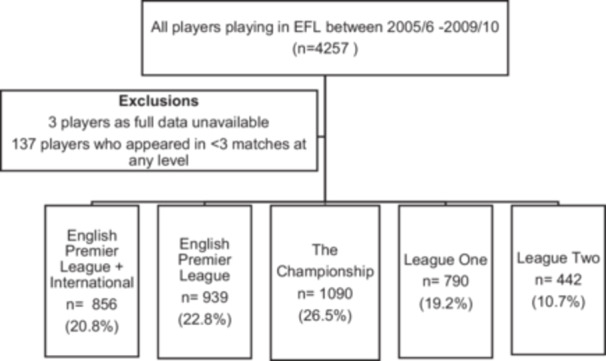
Footballers Included and Ability Groups. EFL, English Football League.

Footballers were categorised into five groups according to the highest level in England in which they made a minimum of three match appearances (Figure 1) and remained in the same group throughout. The groups are English Premier League plus International (EPL + I), EPL alone (EPL), Championship (C), League One (L1) and League Two (L2). To be included in the EPL + I group, a player must have made at least one appearance for their senior national team. Only senior first team players' performance data was included.

The data extracted from the FBREF website contained match appearances, matches started, position, minutes played, and number of full games played throughout an entire senior career. It included participation in English league and cup matches, European Cup matches, and domestic matches played abroad if the player left the UK, but not international matches. Transfers between teams and/or leagues were also noted to determine how this varied over playing careers. Since many footballers play in different countries during their careers, non‐English leagues were matched to their equivalent English level according to www.globalfootballrankings.com in July 2023 (Table 1).

**Table 1:** Overseas clubs matched to English league levels.

English league	Equivalent overseas leagues
Premier League	Bundesliga (DE), LaLiga (ES), Serie A (IT), Ligue 1 (FR)
Championship	Eredivisie (NL), Brasileiro (BR), Primeira Liga (PT), Liga MX (MX), Premier League (RU)
English Football League 1	Super Lig (TR), First Division A (BE), Bundesliga (AT), Superliga (DK), MLS (US), Scottish Premiership, Super League (CH), Primera Division (AR), J1 League (JP), Eliteserien (NO), Ligue 2 (FR), LaLiga 2 (ES), Serie B (IT), Super League (GR), 2. Bundesliga (DE), Allsvenskan (SE)
English Football League 2	A‐League (AU) USL (US), Premier Division (ZA), CSL (CN)
English National League	Any overseas professional league not included above.

The age of the player refers to their age at the start of each season. Career length was defined as the time between first and last professional match. Retirement is determined as the last season in which professional match appearance information in one of the top five English or equivalent leagues is recorded, and retirement age is the age at the end of that season. Playing levels and rates were calculated for each age.

The ‘still playing’ rates of footballers continuing to play that were found were compared with published ‘still playing’ rates after ACLR. The ‘still playing’ rates compared were based on the age at follow‐up which was calculated by adding the number of years post ACLR to the mean age of the cohort studied. They were also compared with the nearest equivalent league levels to those in the published groups.

### Statistical analysis

Statistical analysis was performed using Python, utilising Scipy (version 1.10.1) for general statistical procedures and Lifelines (version 0.27.4) for specialised survival analysis. Descriptive statistics were used to summarise player metrics such as age, position, ability level, match information and career length. T‐ tests were used to compare differences between goalkeepers and outfield players. Analysis of variance (ANOVA) was used to compare differences between multiple groups.

Kaplan–Meier survival curves, which took into account the censoring of players who are still to retire, were generated to predict overall career length, length of time competing at their highest level and to compare goalkeepers with outfield players. The Log‐Rank test was used to predict meaningful differences in career length. Point in time analysis was performed to identify the statistically significant point at which careers diverge. Statistical significance was set at *p* < 0.05.

## RESULTS

The majority of footballers included (1795, 43.6%) competed for a minimum of three matches at EPL level with 47.7% of these playing at least one international game; 26.5% reached C level, 19.2% L1 and 10.7% peaked at L2.

Overall, 339 (8.2%) players are goalkeepers and 3778 (91.8%) are outfield players. These were categorised by the website as defenders, midfield or forwards (Table 2) with 892 (23.6%) players listed as playing in more than one position. The most common dual positions were defender/midfield (444, 11.7%) and forward/midfield (390, 10.3%).

**Table 2:** Player positions.

	EPL + I	EPL	C	L1	L2	All
Goalkeepers (*n*,%)	66 (7.7%)	83 (8.8%)	84 (7.7%)	74 (9.4%)	32 (7.2%)	339 (8.2%)
Defender (*n*, %)	314 (36.7%)	368 (39.1%)	424 (38.9%)	300 (38.0%)	158 (35.8%)	1564 (38.0%)
Midfield (*n*, %)	441 (51.5%)	420 (44.7%)	521 (47.8%)	339 (42.9%)	197 (44.6%)	1918 (46.6%)
Forward (*n*, %)	294 (34.3%)	278 (29.6%)	308 (28.3%)	224 (28.4%)	122 (27.6%)	1266 (29.8%)

*Note*: Due to some players having duplicate positions the total numbers are greater than 4117.

Abbreviations: C, Championship; EPL, Premier League; EPL + I, English Premier League and International; L1, English Football League 1; L2, English Football League 2.

### Actual match appearance metrics

Four hundred and thirty‐five footballers (380 outfield players and 55 goalkeepers) were ‘still playing’ at the censor date of 1 July 2023. Mean career length to date of both these groups was 16 years.

Outfield players started their career and reached their highest level at a younger age on average than goalkeepers (overall start of career 19.9 vs. 21.8 years, *p* =< 0.01, and highest level reached at 22.0 vs. 24.1 years, *p* =< 0.01). Conversely, goalkeepers were able to continue playing until an older age and at their highest level for longer than outfield players (overall 33.3 vs. 30.5 years, *p* =< 0.01, and highest level 28.5 vs. 25.7 years, *p* =< 0.01).

Although the mean overall career length for goalkeepers was longer (12.4 vs. 11.6 years, *p* =< 0.01), there was no significant difference between goalkeepers and outfield players regarding the years spent playing at their highest level (4.4 vs. 4.3 years, *p* = 0.6). The match appearance ages, career lengths, and appearance details between ability groups are shown in Table 3. Defenders played on average significantly more minutes per match appearance, started more games and played more full games than other outfield positions (*p* =< 0.01).

**Table 3:** Appearance information by ability groups.

		Career length	English Premier League & International	English Premier League	Championship	League 1	League 2	All leagues
Outfield players	First appearance age (years, mean, SD)	Overall career	19.6 (2.6)	19.6 (2.7)	20.2 (3.3)	20.2 (3.3)	20.4 (3.3)	20.0 (3.0)
		Highest level	21.9 (3.6)	21.2 (3.6)	22.8 (4.2)	21.8 (4.1)	20.8 (3.5)	21.8 (3.9)
	Last appearance age (years, mean, SD)	Overall career	33.4 (2.8)	32.3 (3.4)	30.5 (4.1)	28.1 (4.8)	25.6 (5.0)	30.5 (4.7)
		Highest level	29.4 (4.1)	25.9 (5.0)	25.5 (4.7)	24.6 (4.9)	23.4 (4.6)	26.0 (5.1)
	Career length (years, mean, SD)	Overall career	14.8 (3.3)	13.7 (3.5)	11.3 (4.1)	8.9 (4.2)	6.2 (4.1)	11.6 (4.7)
		Highest level	7.5 (5.0)	4.4 (3.7)	3.0 (2.5)	3.0 (2.3)	2.9 (2.2)	4.3 (3.8)
	Match appearances (mean, SD)	Overall career	360.9 (139.1)	299.5 (132.0)	267.2 (149.1)	199.4 (143.3)	114.6 (110.9)	264.7 (156.8)
		Highest level	200.3 (161.5)	93.4 (101.9)	73.3 (80.0)	69.5 (74.8)	63.3 (73.5)	103.9 (117.1)
	Matches started (mean, SD)	Overall career	298.7 (127.6)	246.1 (119.5)	221.0 (134.6)	166.2 (130.2)	94.7 (99.1)	218.9 (139.4)
		Highest level	164.5 (142.1)	74.6 (88.8)	57.6 (70.9)	56.4 (66.4)	52.5 (67.2)	84.1 (102.3)
	Full games played (mean, SD)	Overall career	296.2 (126.0)	242.7 (118.3)	217.3 (132.6)	161.0 (128.0)	92.0 (97.1)	215.3 (138.0)
		Highest level	163.7 (140.4)	74.6 (87.5)	57.6 (70.0)	54.4 (65.2)	50.0 (65.6)	83.2 (101.1)
	Minutes per appearance (mean, SD)	Overall career	69.6 (10.6)	67.1 (11.7)	66.3 (13.6)	63.4 (16.1)	61.0 (18.6)	66.0 (14.1)
		Highest level	66.7 (14.9)	59.8 (20.5)	60.9 (19.5)	60.1 (20.3)	59.0 (21.1)	61.5 (19.4)
Goalkeepers	First appearance age (years, mean, SD)	Overall career	21.5 (3.3)	22.3 (4.0)	21.4 (3.8)	22.0 (4.0)	22.4 (3.9)	21.9 (3.9)
		Highest level	24.0 (3.3)	24.5 (4.4)	24.4 (4.5)	23.5 (4.8)	23.0 (4.3)	24.0 (4.3)
	Last appearance age (years, mean, SD)	Overall career	36.6 (3.2)	35.3 (3.8)	33.0 (3.8)	30.5 (5.7)	28.7 (5.1)	33.3 (5.1)
		Highest level	32.8 (4.7)	29.6 (5.1)	27.1 (5.1)	27.0 (6.0)	25.9 (5.7)	28.9 (5.8)
	Career length (years, mean, SD)	Overall career	16.0 (3.7)	14.0 (3.7)	12.5 (3.9)	9.5 (4.8)	7.3 (4.9)	12.5 (4.9)
		Highest level	8.2 (5.3)	4.0 (3.0)	3.1 (3.0)	3.1 (2.6)	3.0 (2.5)	4.4 (3.9)
	Match appearances (mean, SD)	Overall career	382.9 (155.0)	246.6 (116.5)	253.4 (135.1)	182.1 (141.6)	111.0 (126.4)	247.9 (156.5)
		Highest level	212.0 (182.4)	55.5 (63.9)	59.7 (83.5)	64.6 (79.3)	59.6 (73.4)	92.3 (121.1)
	Matches started (mean, SD)	Overall career	381.0 (155.1)	243.7 (116.5)	250.7 (135.0)	180.3 (141.4)	109.5 (126.5)	245.6 (156.4)
		Highest level	210.7 (182.2)	54.2 (63.6)	58.7 (83.1)	63.8 (79.1)	58.6 (73.6)	91.1 (120.9)
	Full games played (mean, SD)	Overall career	380.1 (154.9)	243.5 (115.9)	250.6 (134.8)	180.0 (141.1)	109.1 (126.3)	245.3 (156.0)
		Highest level	210.4 (181.6)	54.1 (63.3)	57.5 (83.0)	63.7 (78.9)	58.5 (73.2)	91.1 (120.5)
	Minutes per appearance (mean, SD)	Overall career	88.2 (2.4)	86.7 (3.6)	86.6 (3.6)	86.2 (4.2)	84.8 (6.7)	86.7 (4.0)
		Highest level	86.7 (9.1)	82.6 (15.8)	82.2 (12.2)	84.4 (10.6)	83.6 (7.6)	83.8 (12.0)
	“Still playing” at censor date (*n*)	Outfield	117	104	110	42	7	380
		Goalkeeper	20	12	16	7	0	55

Abbreviations: C, Championship; EPL, English Premier League; EPL + I, English Premier League & International; L1, League 1; L2, League 2.

### Actual career trajectory

Only 253 (6.1%) footballers played matches at the same league level for their whole careers. 50 (5.8%) EPL + I players only played at the EPL level. Although 143 (32%) of the L2 ability group played solely at that level, their career length was much shorter and lasted less than 3 years in 90 (62.9%) cases.

**Figure 2:**
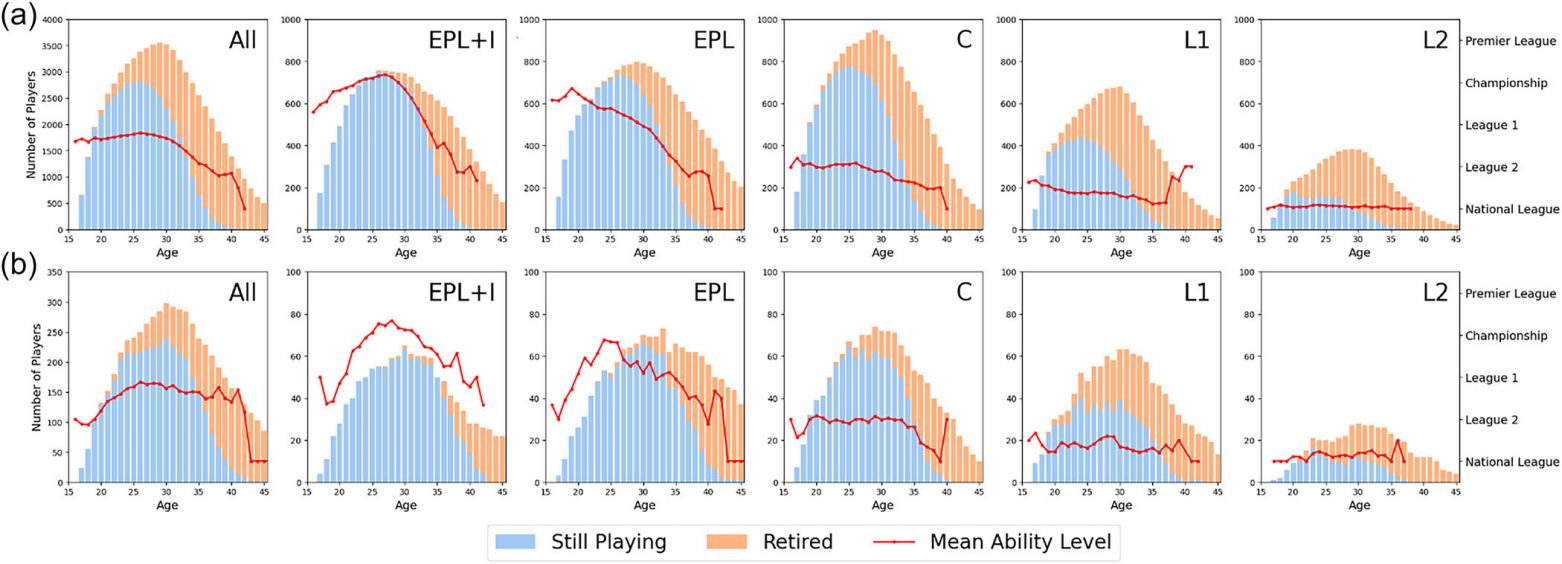
Career trajectory and “still playing” rates by player age. (a) Outfield players. (b) Goalkeepers. C, Championship; EPL, English Premier League; EPL + I, English Premier League and International; L1, English Football League 1; L2, English Football League 2.

The career trajectory graphs (Figure 2), illustrate how footballers' careers change with age and how this varies according to players' abilities. There is a significant difference in the mean length of time outfield footballers play at their highest level according to their level (*p* =< 0.01) with the EPL + I group playing for over 3 years longer (mean 7.5 years) than players in the lower groups (mean 4.4 years in EPL only group, 3.0 years in C and L1 groups and mean 2.9 years in L2).

### Career length survival analysis

**Figure 3:**
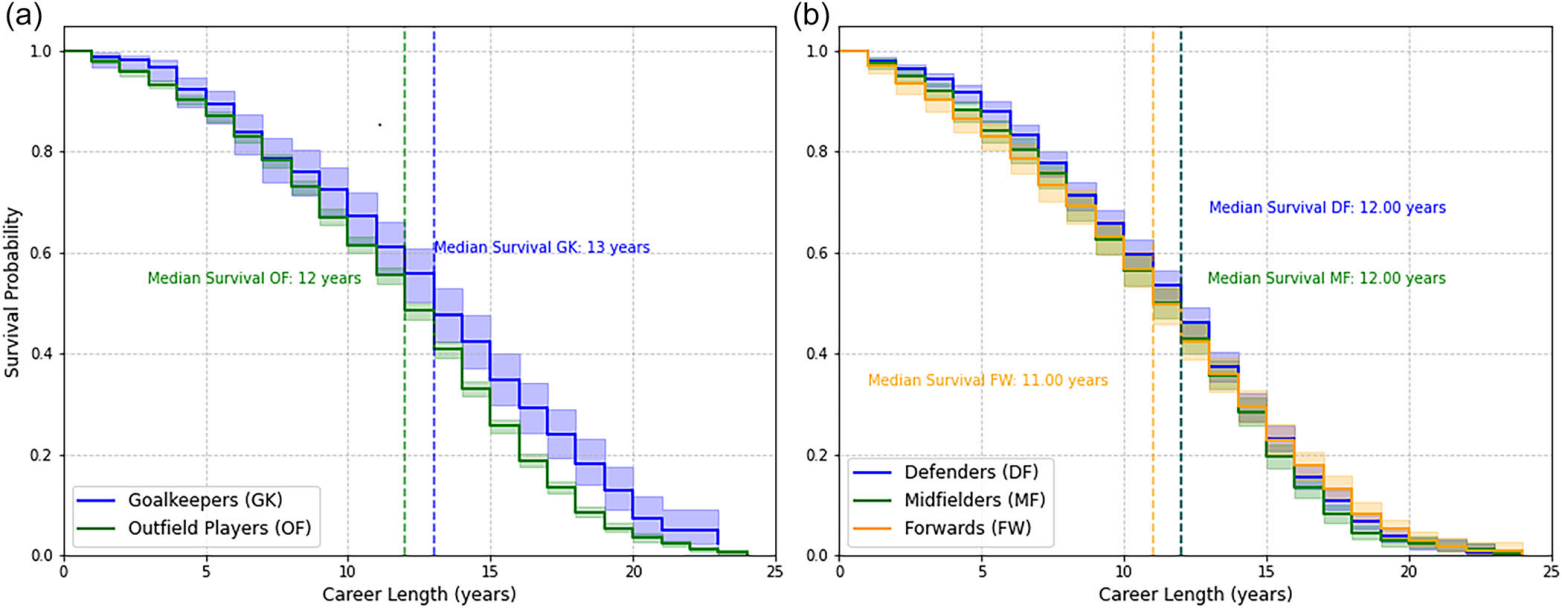
Kaplan–Meier career length survival curves comparing (a) goalkeepers with outfield players and (b) different outfield positions. Probable career length survival varied by ability groups in outfield players (Figure 3, 4) but less so in goalkeepers. Defenders had longer predicted survival (log rank test *p* = 0.01499) than midfielders but there was no significant difference in survival between other field positions.

There was a significant difference (log rank test *p* = <0.001) in overall career length survival times between goalkeepers and outfield players. Survival probability for these groups and for different outfield positions are demonstrated by the Kaplan–Meier survival curves in Figure 3, 4. The ‘point in time divergence’ for these groups (*p* => 0.05) is at 9 years.

**Figure 4:**
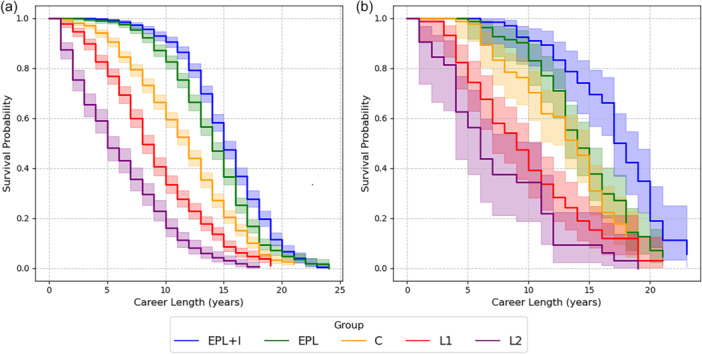
Kaplan–Meier career length survival curves comparing different ability groups for (a) outfield players and (b) goalkeepers. C, Championship; EPL, English Premier League; EPL + I, English Premier League and International; L1, English Football League 1; L2, English Football League 2.

**Figure 5:**
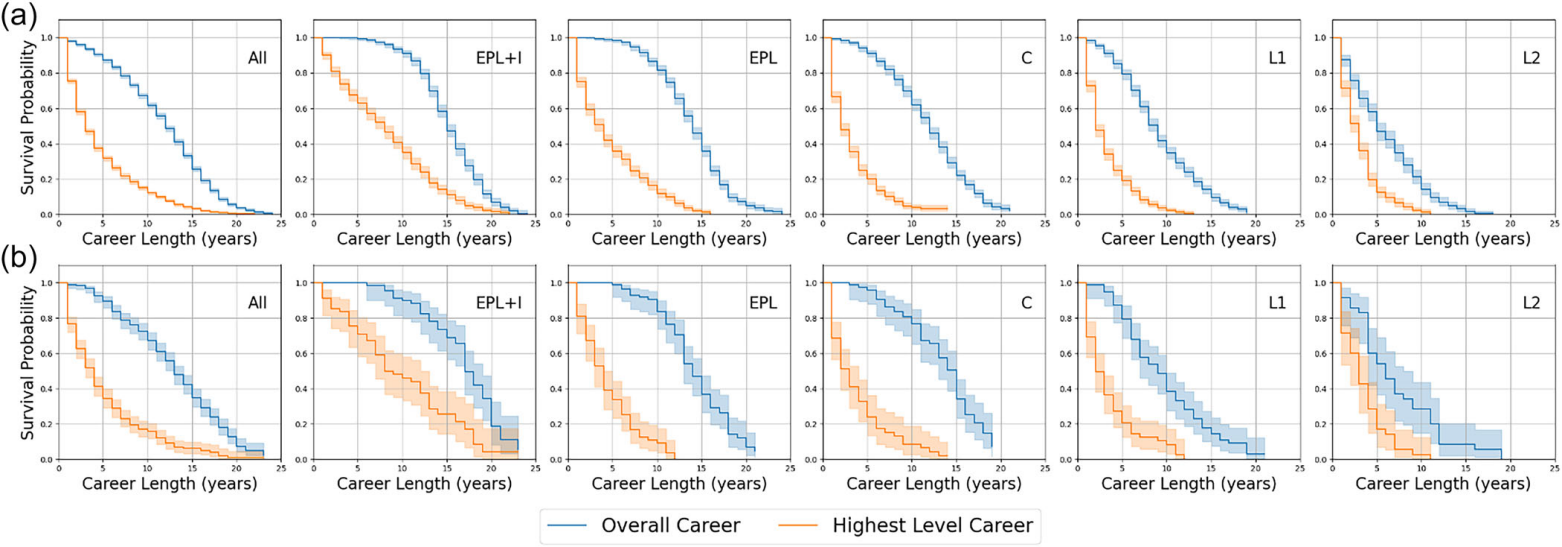
Kaplan–Meier survival curves comparing overall and highest level career lengths by ability group for (a) outfield players and (b) goalkeepers. C, Championship; EPL, English Premier League; EPL + I, English Premier League and International; L1, English Football League 1; L2, English Football League 2.

At 5 years, EPL + I and EPL outfield players have a 60% and 40% probability of continuing to play at their highest level respectively. This compares to a less than 20% chance in the C and lower leagues (Figure 5). At 10 years, the probability of players ‘still playing’ at their highest level is 40% and 18% in the EPL + I and EPL respectively compared with less than 2% in the C or below.

### Comparisons of average career longevity with published career longevity after ACLR

‘Still playing’ rates of the 4117 footballers included in this study categorised by age and ability group are given in Appendix 1. Comparisons between these ‘still playing‘ rates and published career longevity findings after ACLR are demonstrated in Table 4. To determine the ages at which these comparisons were made the number of years post ACLR were added to the mean age of each published cohort.

**Table 4:** Comparison of published “still playing” rates after ACL reconstruction with the average “still playing” rates found in present study.

Literature	League level of cohort in published studies	Comparative leagues in present study	Subject type	Time after ACLR (years)	Mean age (SD) at ACLR (years)	Age at follow‐up (years)	Published ‘still playing’ rates at follow‐up age (%)	Present study ‘still playing’ rates at follow‐up age (%)
Borque et al. [6]	English Premier League and Championship (Male, Post ACL *n* = 82, matched controls *n* = 246)*	English Premier League and Championship	Post ACLR	5	25.2 (±4)	30	84.0**	80.0
				10		35	24.0**	35.0
			Controls	5		30	92.0**	80.0
				10		35	57.0**	35.0
Della Villa et al. [34]	Elite European Professional football (male, *n* = 118)	English Premier League	Post ACLR	4	24.9 (±4.2)	29	69.0	91.2
				5		30	59.7	87.2
Erickson et al. [14]	Major League Soccer (male, *n* = 52)	EFL League 1	Post ACLR	5	25.6 (±4.0)	31	30.8	37.8
Mazza et al. [25]	Top leagues in 8 European countries (male *n* = 195)	English Premier League	Post ACLR	3	25.4 (±3.9)	28	86.4	94.2
Niederer et al. [27]	Top 2 leagues, England, France, Spain, Italy & Germany (male, post ACLR *n* = 132, matched controls n = 264)	English Premier League and Championship	Post ACLR	5	25.3 (±4.2)	30	66.9	79.8
			Controls				80.3	79.8
Pinheiro et al. [28]	Top 5 English leagues and overseas equivalents. (male and female, *n* = 26)	All English leagues	Post Rev ACLR	5	23.9 (±4.3)	29	52.0	71.6
Pinheiro et al. [29]	Top 5 English leagues and overseas equivalents (male, *n* = 200)	All English leagues	Post ACLR	2	24.1 (±4.2)	26	90.0	84.0
				3		27	87.0	80.2
				4		28	81.0	76.2
				5		29	72.0	71.6
Zaffagnini et al. [36]	Italian Professional Footballers (male, *n* = 21)	All English leagues	Post ACLR	4	22.9 (±5.4)	27	71.0	80.2

*Note*: Studies by Arundale et al. [2] and Walden et al. [35] did not include enough age data for comparisons to be made.

Abbreviations: ACL, anterior cruciate ligament; ACLR, anterior cruciate ligament reconstruction; SD, standard deviation.

* 52.5% of post ACL cases 'still playing' at end of study and 65% of matched controls are 'still playing' at end of study.

** Predicted, not actual, 'still playing' rates.

The matched control groups in the studies by Borque et al. [6] and Niederer et al. [27] are the nearest equivalent groups to those in the present study. Borque et al. [6] predicted a ‘still playing’ rate in the EPL and C footballers with mean ages of 30 and 35 years of 92% and 57%, respectively, whereas in the control group in the study by Niederer et al. [27] the actual ‘still playing’ rate at a mean age of 30 was 80.3%. This compared to 79.8% at age 30 in all PL and CC level players in the present study.

In the study by Pinheiro et al. [29], ‘still playing’ rates after ACLR were higher by 6.0%, 6.8%, 4.8%, and 0.4% at 2, 3, 4 and 5 years (ages 26–29 years) respectively when compared with the players from all leagues in the present study. However, Pinheiro also found that 5 year playing rates were lower in those aged 25 or older at surgery, with only 58% ‘still playing’ after ACLR at aged 30 years or older. This compared to 66% of 30 year olds in the current study.

## DISCUSSION

The main findings of this analysis of over 4000 male professional footballers who played in the top four English football leagues are that the average career length is 11.6 years for outfield players and 12.4 for goalkeepers. Furthermore, EPL players, and particularly those who also played for their national teams (EPL + I), have much longer careers, and compete at their highest level for longer than those in lower leagues.

To illustrate the value of this data the present study compared it with the published literature reporting career longevity in professional footballers after ACLR and shows that ‘still playing’ rates in post ACLR players with a mean age of 30 years are up to 12.9% lower than the average “still playing” rates for players of the same age.

Players who compete in both the EPL and internationally (EPL + I) have a 40% probability of playing at their highest level for 10 years compared to 18% if they are playing in the EPL alone (not internationally) and only 2% if they are C level or below. The mean career length of professional outfield players (at any level) is 11.6 years; whilst that of EPL + I players is 14.8 years, reducing to 6.2 years for L2 players.

Hitherto there has been only limited information available regarding career longevity in professional footballers yet, without this information, it is not possible to tell if career longevity is reduced by injury, nor the impact of medical intervention on it. To provide this information, the present study reviewed footballers who were listed as playing in the top four English leagues with careers which spanned a period between 1992 and 2023. This period was chosen as 90% of subjects had retired at the time of the study, thus allowing their full careers to be studied, and yet still be sufficiently contemporary to be relevant to current playing, injury, and treatment patterns.

A systematic review documenting career termination in football found mean career lengths varied between 5.2 and 11 years [7]. Although this is shorter than the average outfield career length in the present study (11.6 years), it covers different leagues in a range of countries. In addition, the present study highlights the different career lengths according to players' level of play (and hence ability) and demonstrates that EPL players, especially those that play at international level, have a career length over twice as long as those who peak at EFL L2. Playing in the top level clubs was also found to be one of the factors delaying retirement age in Portuguese football [26]. Tactical and technical skills have been shown to improve with age [30, 31] and, as these are presumably better in the higher league players, they may be a reason why these players can continue until an older age. In addition, natural selection applies, and ensures only the fittest, strongest, least injury‐prone, and most able athletes make it to the top levels and continue playing for longer.

Age is an important variable that affects career length [6, 20, 31]. The peak performance age of an outfield football player was believed to be 25–27 years with a retirement age of between 31 and 35 years [7, 9, 20, 30]. However there has been a significant increase in the average age (>1.6 years) of elite footballers over the last three decades [20]. Legendary long‐term EPL coaches Alex Ferguson (Manchester United Football Club) and Arsene Wenger (Arsenal Football Club) are both quoted as believing players over 30 to be past their peak [9]. The current data supports this with the average age of players at the last appearance at their highest ability level being 29.4 years in the EPL + I outfield group and 25.9 years in the EPL group. Although unable to continue at their peak level, they continue playing at lower levels to an average age of 33.5 and 32.6 years respectively, which is similar to ages found in other studies [7, 26].

The main reason for investigating career length in footballers was to provide a baseline against which injury and treatment intervention impact can be compared. Several authors [2, 28, 34, 35] have investigated career length after orthopaedic surgeries and have demonstrated that the number of players continuing to play professionally decreases over time. Whether this is due to the natural history of attrition of footballers' careers or due to the injury sustained is not known. Clinicians need evidence of the effectiveness of their treatment to support their recommendations, especially if they are likely to lead to a lengthy absence from competition. Whilst they often have enough evidence to give the player information about RTP times and rates after the recommended treatment [3, 5, 10, 13, 18, 24], they have far less understanding of long‐term effects on their footballers' careers.

Due to a player's natural desire to return to play and their clubs' desire for players to return, clinicians are often under pressure to provide a quick RTP for players so factors such as important matches, or contract negotiations often need to be considered when discussing treatment options with players. The financial implications of top players being unfit for selection are huge: in the UEFA Champions' League clubs (the top few from a European country's top league each year), the market value of a player is estimated to increase by over 435,000 Euros for every season with the club when qualifying for the UEFA Champions' League, and by more than 8 million Euros after winning the competition [20]. Being able to demonstrate that the player can still have a long career after successful surgery and rehabilitation may mitigate their rush to RTP.

The variability in ‘still playing’ rates at different ages and at the different ability (league) levels highlights the importance of ensuring these factors are considered when comparing career longevity after injury and interventions with average career longevity statistics. Matched control groups are the most comparable with the footballers included in the present study, but they report very different ‘still playing’ rates for groups with a mean age of 30 years, (84% and 69%, respectively) [6, 27]. Borque et al. [6] reported predicted rather than actual ‘still playing’ rates, which could account for the 12% difference with the actual ‘still playing’ rates quoted in the present study whereas Niederer et al. [27] reported actual ‘still playing’ rates similar to the current study (80.3% vs. 79.8%).

‘Still playing’ rates of 69.0% and 72% after ACLR at a mean age of 29 years [29, 34] and between 59.7% and 84.0% at 30 years have been reported [6, 27, 34]. This compares with 71.6% of all players in the present study at age 29 and 66.7% at age 30. However this rate varies between 91.2% in the EPL and 27.6% in EFL L2 at age 29 and 87.2% and 22.9%, respectively at age 30, This again highlights the importance of ensuring the players' league levels are considered when comparing average longevity rates.

Three studies [14, 25, 27] reported actual career longevity after ACLR in the EPL and C, (or overseas equivalent level) and found ‘still playing’ rates to be between 4.8% and 12.9% lower than the average ‘still playing’ rates when compared to the equivalent league level in the present study. However, in their study of UEFA league elite footballers Della Villa et al. [34] only included players ‘still playing’ in the top league of each country, rather than ‘still playing’ at any level. The career trajectory graphs (Figure 5) show there is significant difference between the rates of those playing at any level, compared to those playing at the highest level and this is likely to be the reason for the much lower rate in the present study. In studies involving players across several league levels [29, 36] the proportion of high to low league players in the cohort would also affect the “still playing” rates and could account for the differences found between our study and the ones by Pinherio et al. [29] and Zaffagnini et al. [36]. The ‘still playing’ rates were also lower after a study of revision ACLR but as the published cohort also included female footballers it is not a true comparison [28].

By comparing the results of the present study with the reported career longevity outcomes after ACLR it illustrates how the current study's data provides a valuable comparison against which studies reporting the impact of various injuries and their treatments on career longevity can be evaluated. However, when doing so, we have demonstrated that it is important that the age and league levels are considered to ensure accurate comparisons.

There are several limitations to this study, not least that it only includes male footballers. England did not have a fully professional women's football league until 2018 and therefore the historical data needed to include female footballers in this study was not available. The results cannot be used to extrapolate how long female, or any amateur, and recreational footballers will continue to play as it is likely that diverse occupational and social factors would affect these groups differently. Also, although it includes players moving between English and overseas leagues, the results may not be generalisable to leagues in other countries with different playing conditions.

Injury rates for footballers are high [1, 12, 23] and they experience a multitude of different injuries resulting in both short and long term absences from the game. Injury data, even for significant injuries with a long match play absence such as ACL rupture, has been shown to be inaccurate when extracted from public databases [8, 11, 19]. Therefore this study has not considered temporary absences from the game when calculating career length or level played and assumes that these are a normal part of the game and affect all players at all ages and levels. However, it is quite possible that players who have had serious or repeated injuries have shorter than average careers and that, by reporting average career lengths of the entire cohort, including those prone to injury, we underestimate the career lengths of those who remain relatively injury free and overestimate the career length of those post‐injury. Additionally, the fact that some of the players may have undergone ACLR weakens the comparison of the published post ACLR results to this cohort.

Only footballers who had made three or more match appearances were included, which may appear arbitrary. In studies investigating return to sport, the number of match appearances used to determine RTP has varied between 1 and 5 [17, 21, 33]. We felt that one appearance alone was insufficient to truly reflect a player's performance level but, conversely, we did not want to set an excessively high appearance level in case it excluded too many ‘borderline’ players. This decision excluded just 3% of all available footballers between 2005/6 and 2009/10.

We determined retirement age according to the last match appearance at National League level or above. However, it is likely that some players continue to play at semi‐professional or amateur level. It also does not account for players who are still in the squad and fit to play but fail to make selection. Both factors would result in the career length and performance levels being under reported.

When comparing the current study's results with published data we used an exact age whereas the published studies quoted ‘still playing’ rates for the mean age of the cohorts. Given the variation in play rates that can occur within just a few years, the present study can only be considered to give approximate ‘still playing’ comparisons. While a study comparing results with matched controls is likely to be more accurate this is not always feasible or as easy as comparing them with the average figures stated in the present study.

The strength of this study is that it provides career longevity and performance level information from a very large series of professional footballers in a usable format for easy comparisons with outcomes after treatment (Appendix 1). It can be used by those aiming to publish retrospective reviews of a cohort of patients or by a clinician wishing to evaluate their own results.

## CONCLUSIONS

Career duration in footballers is affected by the position played and ability level with goalkeepers and Premier League players having longer careers and remaining at their highest level for longer than players in lower leagues. The average career length for EPL players also playing internationally is 14.8 years compared to 6.2 years in L2. The lower league players have a less than 2% chance of playing at their highest level at 10 years compared to 40% of those playing at both EPL and at international level. Comparisons of the present study's data with the published literature demonstrated that ‘still playing’ rates after ACLR are up to 12.9% lower than the average “still playing” rates in 30 year old professional footballers.

## AUTHOR CONTRIBUTIONS

All authors were involved in initial discussions about the study and provided input as it progressed. Data analysis was performed by Arman Motesharei. The original draft manuscript was written by Mary Jones, with contributions from Arman Motesharei, Simon V. Ball, and Andy Williams. Revisions to the manuscript were made by all authors. All read and approved the final manuscript.

## CONFLICT OF INTEREST STATEMENT

The authors declare no conflicts of statement.

## ETHICS STATEMENT

REC approval was not required as all information used is in the public domain.

## Data Availability

The data that support the findings of this study are openly available at https://fbref.com/en/ Each footballer studied has an individual URL but it is not feasible to list all 4117 URLs.

## Appendix

See Table A1.

**Table A1:** Still playing rates by age and ability groups (outfield and goalkeepers).

	All groups			Premier League and International (PLI)			Premier League (PL)			All premier league			Championship (C)			League 1 (L1)			League 2 (L2)		
**Age**	**All players (*n*)**	**All retired (*n*)**	**All still playing (%)**	**PLI players (*n*)**	**PLI retired (*n*)**	**PLI still playing (%)**	**PL players (*n*)**	**PL retired (*n*)**	**PL still playing (%)**	**PLI & PL players (*n*)**	**PLI & PL retired (*n*)**	**PLI & PL still playing (%)**	**C players (*n*)**	**C retired (*n*)**	**C still playing (%)**	**L1 players (*n*)**	**L1 retired (*n*)**	**L1 still playing (%)**	**L2 players (*n*)**	**L2 retired (*n*)**	**L2 still playing (%)**
**16**	209	0	100.0	55	0	100.0	55	0	100.0	110	0	0	50	0	100.0	30	0	100.0	19	0	100.0
**17**	683	1	99.9	177	0	100.0	158	0	100.0	335	0	0	185	0	100.0	105	0	100.0	58	1	98.3
**18**	1434	13	99.1	320	0	100.0	344	0	100.0	664	0	0	374	2	99.5	268	3	98.9	128	8	93.8
**19**	2059	49	97.6	434	0	100.0	492	0	100.0	926	0	0	543	5	99.1	394	15	96.2	196	29	85.2
**20**	2399	102	95.7	519	0	100.0	569	1	99.8	1088	1	99.9	632	12	98.1	441	36	91.8	238	53	77.7
**21**	2728	173	93.7	628	0	100.0	627	2	99.7	1255	2	99.8	726	22	97.0	491	59	88.0	256	90	64.8
**22**	2953	255	91.4	683	0	100.0	663	4	99.4	1346	4	99.7	793	40	95.0	537	89	83.4	277	122	56.0
**23**	3193	325	89.8	734	0	100.0	723	6	99.2	1457	6	99.6	857	57	93.3	574	116	79.8	305	146	52.1
**24**	3388	389	88.5	775	1	99.9	758	10	98.7	1533	11	99.3	897	71	92.1	626	147	76.5	332	160	51.8
**25**	3498	500	85.7	783	1	99.9	778	19	97.6	1561	20	98.7	938	100	89.3	644	187	71.0	355	193	45.6
**26**	3626	595	83.6	810	4	99.5	817	28	96.6	1627	32	98.0	949	128	86.5	680	226	66.8	370	209	43.5
**27**	3715	737	80.2	808	12	98.5	843	52	93.8	1651	64	96.1	971	164	83.1	702	275	60.8	391	234	40.2
**28**	3791	903	76.2	809	23	97.2	848	73	91.4	1657	96	94.2	1007	219	78.3	727	322	55.7	400	266	33.5
**29**	3830	1087	71.6	806	35	95.7	862	111	87.1	1668	146	91.2	1023	271	73.5	730	374	48.8	409	296	27.6
**30**	3813	1268	66.7	807	56	93.1	861	157	81.8	1668	213	87.2	997	324	67.5	741	417	43.7	407	314	22.9
**31**	3704	1409	62.0	787	85	89.2	844	193	77.1	1631	278	83.0	967	372	61.5	712	443	37.8	394	316	19.8
**32**	3506	1563	55.4	753	109	85.5	817	257	68.5	1570	366	76.7	903	430	52.4	667	464	30.4	366	303	17.2
**33**	3275	1697	48.2	717	180	74.9	794	317	60.1	1511	497	67.1	837	460	45.0	601	456	24.1	326	284	12.9
**34**	3049	1885	38.2	700	266	62.0	746	400	46.4	1446	666	53.9	765	516	32.5	551	441	20.0	287	262	8.7
**35**	2794	1996	28.6	663	356	46.3	708	458	35.3	1371	814	40.6	705	542	23.1	473	411	13.1	245	229	6.5
**36**	2573	2060	19.9	629	438	30.4	672	513	23.7	1301	951	26.9	642	533	17.0	429	385	10.3	201	191	5.0
**37**	2318	2022	12.8	580	456	21.4	621	530	14.7	1201	986	17.9	576	522	9.4	367	344	6.3	174	170	2.3
**38**	2049	1893	7.6	523	460	12.0	583	523	10.3	1106	983	11.1	492	468	4.9	309	301	2.6	142	141	0.7
**39**	1812	1721	5.0	473	432	8.7	533	499	6.4	1006	931	7.5	429	418	2.6	257	252	1.9	120	120	0.0
**40**	1552	1504	3.1	411	388	5.6	492	474	3.7	903	862	4.5	343	339	1.2	205	202	1.5	101	101	0.0
**41**	1314	1294	1.5	351	342	2.6	434	426	1.8	785	768	2.2	273	273	0.0	175	172	1.7	81	81	0.0
**42**	1093	1085	0.7	301	297	1.3	373	370	0.8	674	667	1.0	215	215	0.0	140	139	0.7	64	64	0.0
**43**	888	886	0.2	241	240	0.4	316	315	0.3	557	555	0.4	173	173	0.0	114	114	0.0	44	44	0.0
**44**	720	718	0.3	190	189	0.5	274	273	0.4	464	462	0.4	136	136	0.0	86	86	0.0	34	34	0.0
**45**	586	585	0.2	152	152	0.0	238	237	0.4	390	389	0.3	106	106	0.0	65	65	0.0	25	25	0.0
